# Identifying lipid particle sub-types in live *Caenorhabditis elegans* with two-photon fluorescence lifetime imaging

**DOI:** 10.3389/fchem.2023.1161775

**Published:** 2023-04-13

**Authors:** Wei-Wen Chen, Wenyu Tang, Emily K. Hamerton, Penelope X. Kuo, George A. Lemieux, Kaveh Ashrafi, Marcus T. Cicerone

**Affiliations:** ^1^ School of Chemistry and Biochemistry, Georgia Institute of Technology, Atlanta, GA, United States; ^2^ School of Environmental and Biological Sciences, Rutgers University, New Brunswick, NJ, United States; ^3^ School of Medicine, University of California, San Francisco, CA, United States

**Keywords:** *C. elegans*, lipid metabolism, aging, yolk lipoprotein, two-photon excitation fluorescence (TPEF) imaging, coherent Raman imaging, *in vivo* imaging, fluorescence lifetime imaging microscopy (FLIM)

## Abstract

Fat metabolism is an important modifier of aging and longevity in *Caenorhabditis elegans*. Given the anatomy and hermaphroditic nature of *C. elegans*, a major challenge is to distinguish fats that serve the energetic needs of the parent from those that are allocated to the progeny. Broadband coherent anti-Stokes Raman scattering (BCARS) microscopy has revealed that the composition and dynamics of lipid particles are heterogeneous both within and between different tissues of this organism. Using BCARS, we have previously succeeded in distinguishing lipid-rich particles that serve as energetic reservoirs of the parent from those that are destined for the progeny. While BCARS microscopy produces high-resolution images with very high information content, it is not yet a widely available platform. Here we report a new approach combining the lipophilic vital dye Nile Red and two-photon fluorescence lifetime imaging microscopy (2p-FLIM) for the *in vivo* discrimination of lipid particle sub-types. While it is widely accepted that Nile Red staining yields unreliable results for detecting lipid structures in live *C. elegans* due to strong interference of autofluorescence and non-specific staining signals, our results show that simple FLIM phasor analysis can effectively separate those signals and is capable of differentiating the non-polar lipid-dominant (lipid-storage), polar lipid-dominant (yolk lipoprotein) particles, and the intermediates that have been observed using BCARS microscopy. An advantage of this approach is that images can be acquired using common, commercially available 2p-FLIM systems within about 10% of the time required to generate a BCARS image. Our work provides a novel, broadly accessible approach for analyzing lipid-containing structures in a complex, live whole organism context.

## 1 Introduction

*Caenorhabditis elegans* (*C. elegans*) is one of the most heavily utilized model systems for elucidating the molecular mechanisms of organismal longevity and health span. Most of the pathways that have been found to affect aging, also affect fat metabolism, suggesting that a close connection exists between lipid metabolism and aging (Bitto et al., 2015; Lemieux and Ashrafi, 2016). Most of these pathways have evolutionarily conserved counterparts in other species including humans (Lemieux and Ashrafi, 2015b; Watts and Ristow, 2017). Almost all studies of fat regulation and aging in *C. elegans* are conducted on hermaphrodites, which are capable of producing a large number of progeny. Compared to mammals, *C. elegans* has a simplified anatomy and lacks adipocytes. Lipid-rich particles have been noted in several tissues of *C. elegans*, most notably its intestinal and epidermal tissues (Hellerer et al., 2007). By virtue of its size, the intestine contains a majority of *C. elegans* lipids and most studies of *C. elegans* fat content have treated the intestinal lipid depots as analogous to the lipid depots found in mammalian adipocytes. However, the intestines of *C. elegans* hermaphrodites are the production site of lipid-rich particles, named yolk, which transfer lipids from the parent to the developing progeny (Grant and Hirsh, 1999; Perez and Lehner, 2019). Thus, accurate analysis of fat mass or adiposity requires the ability to distinguish between lipid reservoirs that can be mobilized to meet the somatic energetic demands of the parent from those that are destined to the progeny.

The transparent, microscopic nature of *C. elegans* make vital, microscopy-based assays valuable in this organism. Broadband coherent anti-Stokes Raman scattering (BCARS) microscopy is particularly well suited to this endeavor given its ability to image intrinsic, sub-cellular components directly and quantitatively in live specimens based on the chemical differences shown in the entire biological-relevant Raman spectral range (600–3,100 cm^−1^). Our studies using this technique in *C. elegans* revealed that the lipid-containing structures of the intestine are highly heterogeneous in chemical composition with varying degrees of unsaturation, protein content, and whose lipid contents varied significantly in half-life (Chen et al., 2020). Broadly, three classes of lipid-rich particles were observed. The most protein-rich lipid-containing structures spectroscopically resemble yolk particles. Consistent with this idea, these particles are absent in male *C. elegans* which do not produce yolk. These yolk particles have a fast turnover rate, partially colocalize with the yolk-protein VIT-2, and are continuously transported from the intestinal cells of gravid adults to their developing oocytes in the germline. A second class of lipid-rich particles observed were those that, relative to the yolk particles, were protein-poor, also present in males, and exhibited a relatively slow turnover under conditions where animals have ample access to food but could be mobilized upon long-term fasting. The characteristics of this subset of particles suggest that they function as energy storage particles. Interestingly, these lipid-storage particles, while found in the intestinal cells, are the dominant lipid-rich particles found in the epidermal cells. Finally, BCARS analyses also identified lipid-rich particles with intermediate characteristics between those of lipid-storage and yolk particles. Notably, upon brief starvation particles with lipid-storage characteristics of the intestine and the epidermis behave differently. Within the intestine, the storage particles are converted into intermediate and yolk particles, while within the epidermis, these particles persist for long periods and gradually diminish without converting into yolk. Thus, within the adult hermaphrodite intestine, the vast majority of lipid particles are likely in service of sending lipids to the progeny while the majority of lipid-rich particles of the epidermis appear to have functions similar to energetic reservoirs used for somatic maintenance (Chen et al., 2020).

Routine detection and measurement of the fat content of *C. elegans* rely either on histochemical methods, such as applications of Oil Red-O, Bodipy labeled fatty acids, LipidTOX, and Sudan Black B to fixed *C. elegans* or on biochemical measurements of lipids extracted from whole animal lysates (Elle et al., 2010). The histochemical methods can suffer from potential artifacts caused by the fixation process (Fukumoto and Fujimoto, 2002; Lemieux and Ashrafi, 2015a; Chen et al., 2020) while anatomical information is lost in the biochemical methods. A further problem is that, unlike BCARS, none of the existing histochemical and biochemical methods can distinguish fats that are in storage depots from fats that are contained in various lipoprotein particles. To address these issues, protein markers have been used as a strategy for discriminating between yolk particles and triglyceride stores. For example, VIT-2::GFP is commonly used as a marker of yolk given the association of vitellogenins with yolk (Grant and Hirsh, 1999) and DHS-3::GFP is considered a marker of lipid droplets (Zhang et al., 2012). However, these protein markers only detect a subset of lipid-rich particles, and their assessment is at best an indirect measure of the nature of the lipid content of these particles (Chen et al., 2020). While BCARS microscopy allowed for unambiguous identification of various lipid-rich particles in *C. elegans*, it requires expensive, custom-built systems with a high technical threshold to build and operate. Additionally, because BCARS images are very high content, image acquisition and processing pipelines using this system are yet sufficiently time-efficient for investigating a large number of conditions typical for *C. elegans* studies. Thus, there is an unmet need for a methodology that, similar to BCARS, preserves anatomical information and can distinguish between the different lipid particles of *C. elegans* but is more broadly accessible and available than BCARS.

Fluorescence lifetime imaging microscopy (FLIM) collects the fluorescence decay signals emitted from fluorophores that are excited by a time-modulated light. The same fluorophore could yield different fluorescence lifetimes depending on its structural conformation, binding interactions, or local environment (Berezin and Achilefu, 2010; Becker, 2012), providing an extra dimension of contrast in fluorescence imaging. FLIM has been successfully applied to solve autofluorescence problems in cell and tissue imaging (König, 2020), unmix multiple stains with similar emission spectra (Vu et al., 2022), study cell mitochondrial redox states (Walsh et al., 2021), as well as investigate the changes in the molecular environment such as pH (Lin et al., 2003), viscosity (Kuimova et al., 2008), polarity (Wang et al., 2015), and concentration of ionic species (Despa et al., 2000; Wilms and Eilers, 2007). The recent development of the fit-free FLIM phasor approach, a fast time-frequency domain conversion of FLIM data, greatly improves the speed of data analysis (Digman et al., 2008; Stringari et al., 2011; Ranjit et al., 2018), making it possible to achieve a parallel multichannel detection scheme followed by fast multidimensional phasor data analysis (Scipioni et al., 2021).

Here we present an approach combining the lipophilic dye Nile Red and two-photon fluorescence lifetime imaging microscopy (2p-FLIM) to differentiate lipid particle sub-types in live *C. elegans*. We show that, at enhanced Nile Red concentrations (5 *μ*M), lipid structures become evident with vital staining. We further develop a Nile Red 2p-FLIM measurement and analysis pipeline that can effectively differentiate *C. elegans* lipid particles with performance similar to BCARS but in a fraction (≤10%) of the time required for BCARS acquisition and analysis using a widely available commercial microscope. This work provides a potentially widely accessible approach for examining lipid sub-types in live *C. elegans* for fat regulation and aging-related studies.

## 2 Materials and methods

### 2.1 Strains and reagents

The *C. elegans* strains used in this study included wild-type (Bristol N2), LIU1 *ldrIs1* [*dhs-3p::dhs-3::GFP + unc-76(+)*], DH1033 *bIs1* [*vit-2p::vit-2::GFP + rol-6(su1006)*], and CB1370 *daf-2(e1370)*. All of the strains were obtained from the *Caenorhabditis* Genetics Center (CGC), University of Minnesota. Nematodes for measurements were synchronized by the two-generation egg laying method. Synchronized worms were maintained on nematode growth media (NGM) plates, seeded with *Escherichia coli* strain OP50 as bacterial food source, and incubated at 20°C.

### 2.2 Nile Red vital staining

Nile Red (Sigma-Aldrich) was added in DMSO to a final concentration of 5 mM as stock solution. Sonication could be applied to help Nile Red dissolution. All Nile Red OP50 plates were freshly prepared before staining. For preparing the Nile Red OP50 plates, the OP50 slurry was mixed well with Nile Red and spread on unseeded 6-cm NGM plates. Then, the OP50 plates containing Nile Red (with 50 nM or 5 *μ*M final concentration) were stored away from light at room temperature overnight to allow bacterial growth. Subsequently, 30–50 synchronized worms were transferred to the OP50 plates containing Nile Red and incubated at 20°C for 4 h or overnight until measurements. Non-polar (neutral) lipid solution (Cayman) was used for *in vitro* measurement. Sodium azide (Sigma-Aldrich) was used to anesthetize worms for microscopy.

### 2.3 Lifespan assay

The lifespan assay was initiated with six plates of synchronized 1-day adult wild-type *C. elegans* (*n* = 30 per plate). Three of the six plates were previously grown overnight on 6-cm OP50 plates containing Nile Red with 5 *μ*M final concentration. These Nile-Red-stained worms were then transferred to fresh OP50 plates without Nile Red. Nematodes in all six plates were transferred to newly seeded OP50 plates every day for the first 10 days of adulthood until no more fertilized eggs were observed. The total number, death number, and censured number of worms in six plates were recorded respectively every 2–4 days until all were dead. The censored number accounted for the missing worms and those dead or injured during transfer. The survival rates of the worms with and without Nile Red overnight feeding were calculated by Kaplan–Meier estimator. The statistical significance of Nile red toxicity on lifespan was estimated by Log-rank test.

### 2.4 BCARS and two-photon fluorescence lifetime imaging microscopy

The BCARS setup is as previously described (Chen et al., 2020). The two-photon fluorescence lifetime imaging microscopy (2p-FLIM) measurements were performed on a Bruker Ultima Investigator microscope (Bruker, Billerica, MA) with two Hamamatsu H10770 Photomultiplier tubes (Hamamatsu Photonics, Shizuoka, Japan) for detection and a time-correlated single photon counting (TCSPC) card (SPC-150 card, Becker *&* Hickl, GmbH, Berlin, Germany) for FLIM data acquisition. A 100-fs pulse laser with an 80 MHz repetition rate (Discovery Chameleon, Coherent, Santa Clara, CA) was used as the two-photon fluorescence excitation source. The excitation wavelength for both Nile Red and GFP was set at 920 nm. A water immersion 40X/0.8 W microscope objective (Nikon, Tokyo, Japan) was used for imaging. A 565 nm long-pass dichroic mirror (Chroma Technology Corporation, Bellows Falls, VT) and a 758 nm short-pass filter (Semrock, Rochester, NY) were put in the collection path to reject the excitation laser pulses. The typical acquired image size was 1,024 × 1,024 pixels (about 166 × 166 *μ*m^2^ field of view) and with a 0.5 ms pixel dwell time.

### 2.5 Calculation of the overlap between Nile Red and DHS-3::GFP signals

Both Nile Red and DHS-3::GFP images were processed with the same procedure. A mild Gaussian filter (sigma set to 1 pixel) was first applied to decrease the granularity of the particles on the image to have more uniform lipid structures. Then, the rolling-ball algorithm (Sternberg, 1983) with the ball diameter set to 60 pixels was used for background removal. The ball diameter (60 pixels, which is about 9.6 *μ*m) was chosen to be slightly bigger than the largest lipid particles observed in the Nile Red images so that the lipid structures in the images can be preserved after background removal. After removing the background, the image was thresholded with Otsu algorithm (a popular method for image thresholding) (Otsu, 1979) and converted into a binary image for image segmentation. Finally, the overlapping area between the binary images of Nile Red and DHS-3::GFP was calculated.

### 2.6 Data processing and machine-learning analysis

The BCARS ensemble machine-learning analysis pipeline can be found in (Chen et al., 2020). For 2p-FLIM data conversion, each pixel containing 256 time bins as the fluorescence decay curve was calculated using Eqs 1, 2, and converted to its phasor components (*g* and *s*). The SHG signal of a thin ZnSe film was used as the IRF reference (with ∼0 ns lifetime) for the phase and amplitude correction for converted phasor components. For the 2p-FLIM ensemble machine-learning analysis, we first took GFP and Nile Red images, applied the same image process protocol described in Section 2.5 to identify the particles with both Nile Red and GFP signals, and used those DHS-3::GPF-positive and VIT-2::GPF-positive Nile Red particles as the ground-truth for non-polar lipid-dominant and polar lipid-dominant particles respectively. We used low-concentration Nile Red vital staining images that contained non-specific stained particles for the non-specific staining ground-truth images. After that, six images associated with the FLIM data including fluorescence intensity, phasor *g* and *s*, as well as the HSV-RGB converted three-channel (R, G, B) images were stacked into a 6D matrix. Then, descriptors including the median, mean, standard deviation, maximum, minimum, kurtosis of histogram distribution, skewness of histogram distribution, and first 3 order moments of the histogram were calculated, for each particle in the 6D matrix were calculated (10 descriptors × 6 images). The area, Feret’s diameter, perimeter, and integrated pixel intensity of each particle were also calculated, resulting in a total of 64 descriptors to describe the FLIM and morphological properties of each particle. The 64 descriptors for each particle were then subjected to ensemble machine-learning training and classification shown in Section 3.4. About 6,000 particles in those grand-truth images were used as the training data set. We randomly chose at least 1,500 particles from one class and randomly chose at least 2,000 particles from the other two classes for training individual binary classification classifiers. In each training process, 80% of the particles were used for the classification training and the rest for the validation. A soft voting rule (or weighted classifier output) was adopted for the ensemble classification training. The fine-tuning of the weighting factors was done by iterating the training process at least 10 times until reaching a stable precision. Finally, the validation result showed ∼90% precision for the ensemble classification. Python was used for data processing, image analysis, and statistics. Image and statistical analysis were performed using scikit-learn (v 1.2.0) and scikit-image (v 0.19.3) under Python v 3.9.

## 3 Results

### 3.1 *In vivo* imaging of *C elegans* fats with Nile Red staining

**FIGURE 1:**
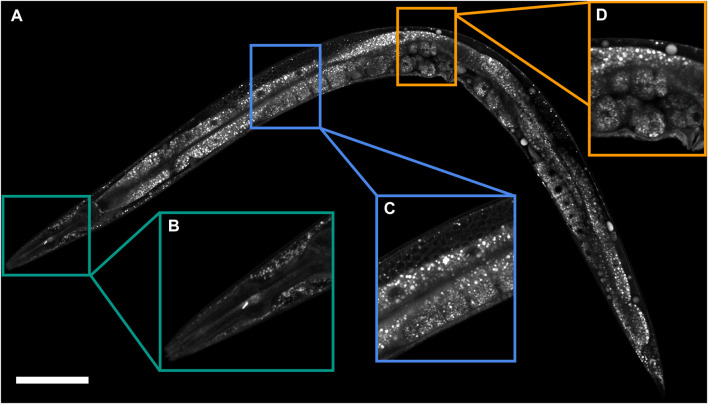
Lipid structures revealed by Nile Red 2p-FLIM. **(A)** 2p-FLIM image of a Nile-Red-stained 1-day adult wild-type worm. **(B–D)** The expansion region of the head, intestine/gonad, and embryos, respectively. Scale bar, 100 *μ*m.

The Nile Red fluorescence intensity increases about two orders of magnitude when binding to lipids (Greenspan et al., 1985), providing high contrast for the lipid-rich structures. While Nile Red is frequently used for fixative staining, we identified that the lipid structures in live *C. elegans* can be stained by application of Nile Red at 5 *μ*M final concentration, a higher concentration compared to previous applications of Nile Red as a vital dye, with 4 h –8 h staining time (Figure 1; Supplementary Figures S1A, C. See section *Materials and Methods* for staining details). In the anesthetized, live, Nile-Red-stained *C. elegans*, we observed many structures in the skin-like epidermis near the pharynx (Figure 1B), intestine, and gonad (Figure 1C), as well as in embryos (Figure 1D). The distribution, pattern, and size of those bright spots were essentially identical to those lipid structures captured by coherent Raman imaging (Hellerer et al., 2007; Le et al., 2010; Yen et al., 2010; Klapper et al., 2011; Wang et al., 2011; 2014; Fu et al., 2014; Chen et al., 2016; Shi et al., 2018; Chen et al., 2020). The size distribution of Nile-Red-tagged particles (Supplementary Figure S1B) was similar to previous BCARS *in vivo* measurements of lipid-rich particles (Chen et al., 2020), both showing a peak around 2 *μ*m and with largest size close to 7*μ*m, suggesting that most of the Nile-Red-tagged particles were lipid-rich particles. Furthermore, similar to BCARS observation (Chen et al., 2020), no lipid fusion was observed during Nile Red 2p-FLIM *in vivo* imaging. Although this method used a high concentration of Nile Red, no appreciable change was observed in the lifespan of the worms with overnight staining (Supplementary Figure S2).

We next investigated the contribution of autofluorescence because its emission spectrum (480 nm –680 nm) (Mak, 2013) is highly overlapped with Nile Red’s (530 nm –700 nm, depending on the molecular environment) (Greenspan et al., 1985). We measured the fluorescence intensity from 1-day adult wild-type worms with and without Nile Red vital staining. We found the Nile Red signal was on average two orders of magnitude stronger than autofluorescence under the same laser excitation wavelength, power, detection condition, and pixel dwell time (Supplementary Figures S1C–F), suggesting that the contribution of autofluorescence signal is negligibly small. The histograms (Supplementary Figures S1C, E) provided a reference for determining an intensity threshold where pixels below this threshold were considered indistinguishable from autofluorescence and thus were excluded in the phasor analysis.

### 3.2 Environment-sensitive Nile Red 2p-FLIM signals characterized *in vivo* and *in vitro*

**FIGURE 2:**
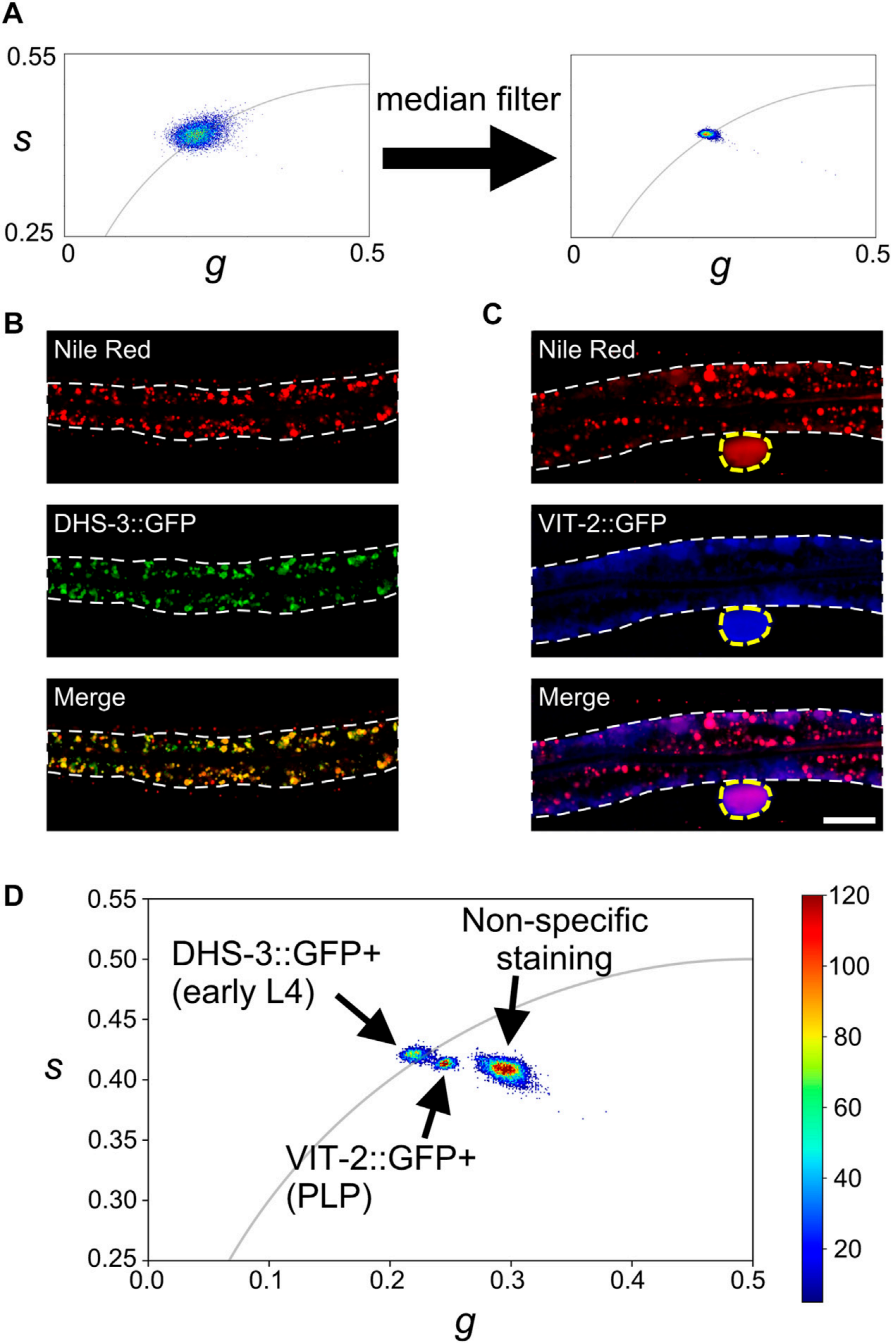
Phasor analysis of Nile Red 2p-FLIM signals. **(A)** The pixel FLIM phasor plots before and after applying a median filter to *s* and *g* images. **(B** ,**C)** The Nile Red (upper), GFP (middle), and merge (lower) images of a DHS-3::GFP transgenic early L4 worm and a VIT-2::GFP transgenic adult worm, respectively. The white dash lines indicate the intestine, and the yellow dash line in **(C)** indicates the pseudocoelomic lipoprotein particle (PLP) that is used for the phasor analysis. Scale bar, 20 *μ*m. **(D)** The phasor plot of Nile Red signal in the DHS-3::GFP-positive (in early L4 worms), VIT-2::GFP-positive (PLP), and non-specific staining (low-concentration Nile Red vital staining of wild-type adult worms) regions. The universal semicircle (in gray) indicates the single-component lifetimes that can be fitted with single exponential decay curves. The color bar represents pixel counts.

The lifetime information was sampled in 256 time bins for each pixel in the FLIM raw data, e.g., time-correlated single-photon counting histogram. The acquired three-dimensional FLIM data set (x pixels, y pixels, time bins) was then transferred to phasor components using the following relations (Weber, 1981; Ranjit et al., 2018):

$$g_{i,j}\left(\omega \right)=\int_{0}^{T}I\left(t\right)\cdot \cos\left(n\omega t\right)dt/\int_{0}^{T}I\left(t\right)dt$$ (1)

$$s_{i,j}\left(\omega \right)=\int_{0}^{T}I\left(t\right)\cdot \sin\left(n\omega t\right)dt/\int_{0}^{T}I\left(t\right)dt$$ (2)

where *I(t)* is the fluorescence decay in time domain; *g*_*i*,*j*_ and *s*_*i*,*j*_ are the x and y coordinates of the phasor plot; *ω* is 2*πν*, where *ν* is the laser repetition rate; *n*, is the harmonic of *ν* chosen (*n* = 1), and *T* = 1/*ν*. After conversion, a 5 × 5 median convolution filter was applied to sharpen the border of the converted phasor plot, decreasing the variance of phasor distribution (Figure 2A), as described in reference (Ranjit et al., 2018). The advantage of this approach is that the median filter is only applied to the phasor plot so it does not affect the spatial resolution of the image.

As a reference point, we imaged transgenic worms that express DHS-3::GFP and VIT-2::GFP (Figures 2B–D) with high-concentration (5 *μ*M) Nile Red vital staining, the worm with low-concentration (50 nM) Nile Red vital staining (Supplementary Figure S3 left), as well as Nile Red in water and lipid solution as an *in vitro* reference (Supplementary Figure S4A). Most of the lipid-storage particles in the L4-stage intestine are ringed by DHS-3::GFP (Chen et al., 2020). We thus performed Nile Red vital staining of transgenic worms that express DHS-3::GFP at early L4 stage for 2p-FLIM as a reference point. We found a high overlap between DHS-3::GFP and Nile Red signals in the early L4 stage (Figure 2B). Most (91.4%) of the Nile Red pixels in the intestine were DHS-3::GFP-positive (see section *Materials and Methods* for calculation details). While the DHS-3::GFP signal was only expressed in the intestine, Nile Red can label lipid particles in the epidermis, intestine, gonad, and embryos (Figures 1, 2B).

We next examined transgenic worms that express VIT-2::GFP. The VIT-2::GFP marker may only be suitable for detecting yolk particles just prior to secretion because its signal is largely restricted to the basolateral surface of the intestine. In the pseudocoelom, the VIT-2::GFP signal co-localizes with the BCARS lipid signal (Chen et al., 2020). We found that while the VIT-2::GFP signal partially overlapped with Nile-Red-stained particles in the intestine, the pseudocoelomic lipoprotein particle (or PLP) was precisely tagged by both Nile Red and VIT-2::GFP (Figure 2C). Notably, we observed the delipidation of VIT-2::GFP-containing particles in embryos with NR 2p-FLIM (separate GFP and Nile Red signals in the embryos shown in Supplementary Figure S5), in agreement with the previous BCARS finding (Chen et al., 2020).

Under low-concentration Nile Red vital staining conditions, most of the Nile Red molecules have been reported to label acidic gut granules rather than lipid particles in the intestine (O’Rourke et al., 2009; Yen et al., 2010). Our microscope settings yielded mostly homogeneous staining in the intestine, with some muted punctate regions. It is possible that these could be the acidic gut granules, however, we did not detect appreciable Nile Red signals even in the punctuate regions (Supplementary Figure S3). Finally, we pooled these reference data and presented them on the same plot. The results show that the three components, Nile Red signals in the DHS-3::GFP-positive (in early L4 stage), VIT-2::GFP-positive (in PLP), and non-specific staining region (low-concentration Nile Red vital staining), are well-segregated (Figure 2D; Supplementary Figures S4B–D). This clearly indicates that the Nile Red 2p-FLIM possesses the resolution to differentiate non-specific staining as well as the two lipid particle sub-types.

Nile Red is sensitive to the type of solvent in the micro-environment as its fluorescence quantum yield and lifetime decreases markedly with the increase of hydrogen bonds (Cser et al., 2002). When in water solution, small Nile Red aggregates form (Ray et al., 2019), resulting in measured phasor cluster (Supplementary Figure S4A) shifting toward a shorter lifetime (lifetime = 0 ns when *g* = 1 and *s* = 0). Our FLIM data obtained from low-concentration Nile-Red-stained worms similarly showed a shifted phasor cluster (Figure 2D; Supplementary Figure S4). In a lipid-rich environment such as in a non-polar lipid solution, the Nile Red phasor cluster (Supplementary Figure S4A) located on the universal semicircle (gray curve) whose lifetimes on this semicircle can be perfectly described as single exponential decays. Our FLIM results suggest that the Nile-Red-stained, DHS-3::GFP-positive lipid particles are single-component-dominant because their *g* and *s* phasor components locate around on the universal semicircle, while VIT-2::GFP-positive particles may contain multiple components (Figure 2D; Supplementary Figures S4B, D). Compared with the *in vitro* measurement, the center of DHS-3::GFP-positive phasor cluster was located at almost the same position of the phasor cluster of Nile Red dissolved in non-polar lipids (Supplementary Figure S4A). These results are in agreement with the fact that the two lipid particle types are chemically distinct. From Raman and chromatographic analysis results, the lipid-storage particles are non-polar lipid-dominant, protein-poor, and with triacylglycerol/phospholipid ratio (TAG/PL) ∼20, while the yolk particles contain more polar lipids, more protein components, and with TAG/PL ∼0.5 (Kubagawa et al., 2006; Vrablik et al., 2015; Chen et al., 2020).

### 3.3 FLIM color representation

**FIGURE 3:**
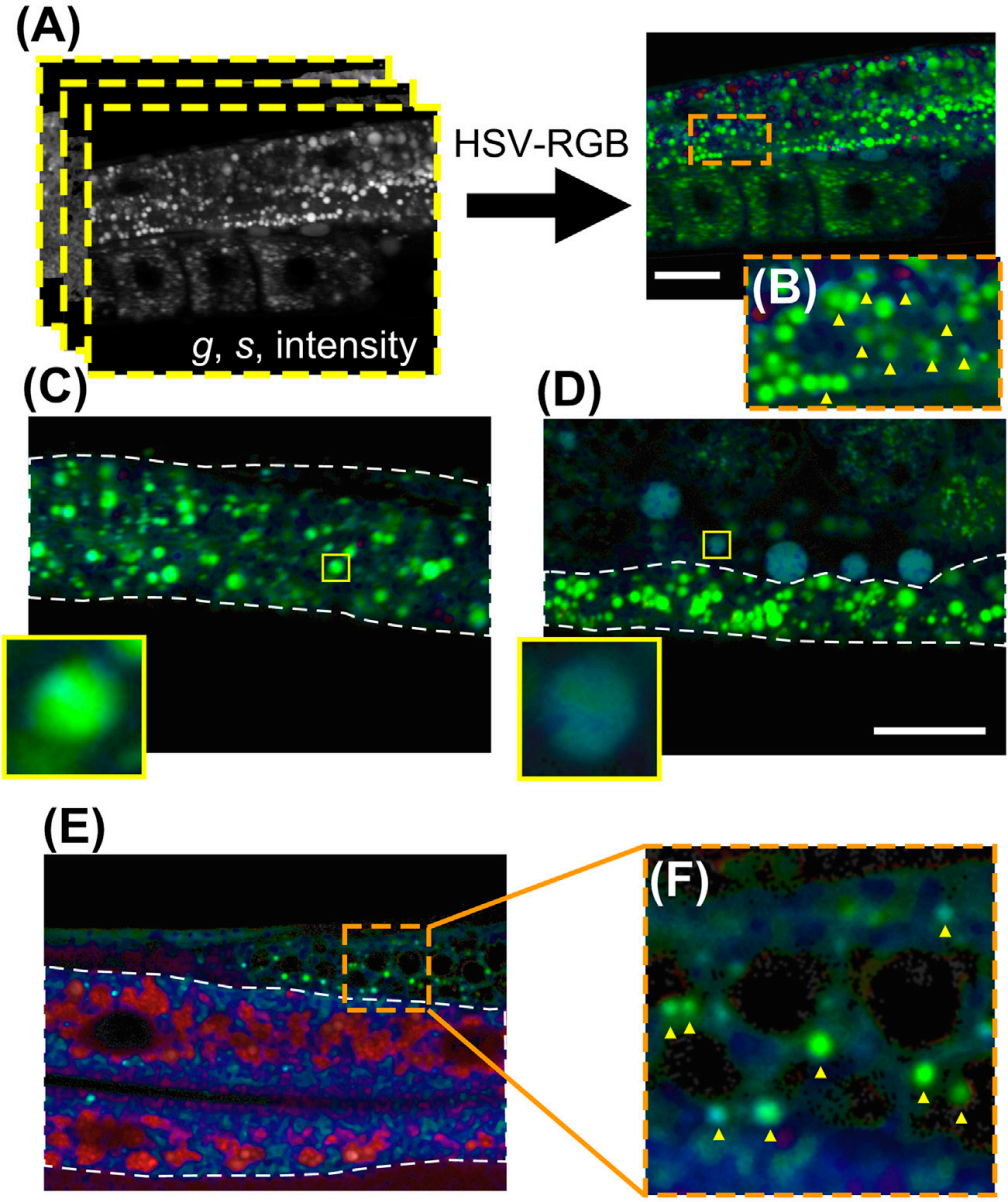
FLIM color representation. **(A)** Visualization of FLIM phasor data of a wild-type adult worm through HSV-RGB transformation. **(B)** The expansion image of the orange box is shown in **(A)**. **(C)** The FLIM color representation of an early L4 DHS-3::GFP transgenic worm stained with high-concentration Nile Red, where the inset is the expansion of a lipid-storage particle. **(D)** The FLIM color representation of an adult VIT-2::GFP transgenic worm stained with high-concentration Nile Red, where the inset is the expansion of a pseudocoelomic lipoprotein particle (PLP). **(E)** FLIM color representation of an adult wild-type worm stained with low-concentration Nile Red. **(F)** The expansion of the orange box in **(E)**. White dashed lines indicate intestine. Scale bar, 20 *μ*m.

In order to better visualize the acquired 2p-FLIM data, we performed HSV-RGB transformation. The converted *g* and *s* phasor images, as well as fluorescence intensity image, were stacked into a three-dimensional matrix as the “H” (hue), “S” (saturation), and “V” (value) components, respectively. Then, the HSV matrix was transformed into the color space as an RGB image (Figure 3A). The lifetime difference thus was converted to the change in colors without any fitting. After HSV-RGB transformation, the non-polar lipid-dominant lipid-storage particles were green or yellow-green color (Figure 3C), while the polar lipid-dominant yolk particles were in cyan or dark cyan (Figure 3D). More lipid particles with a color varying between green and cyan were observed in wild-type worms (examples of which are indicated by arrowheads in Figure 3B). Many lipid particles in the skin-like epidermis near the pharynx are yellow-green color after HSV-RGB transformation (Supplementary Figure S6), indicating most of these epidermal particles are lipid-storage-like particles, in agreement with the previous BCARS results (Chen et al., 2020).

In *C. elegans* with low-concentration Nile Red vital staining, predominant pixels in the intestine are shown in red or purple (Figure 3E). The fluorescence intensity of those pixels was weak compared to high-concentration Nile Red staining (Supplementary Figure S3), although some small lipid particles can be still observed in the gonad region (some of which are indicated by yellow arrowheads in Figure 3F). These results demonstrate that the high-concentration Nile Red vital staining can effectively label intestinal lipid particles, and the FLIM phasor analysis and color representation further yields a direct visualization of FLIM components, facilitating rapid recognition of lipid particle sub-types.

### 3.4 Quantitative analysis of lipid particle sub-types

**FIGURE 4:**
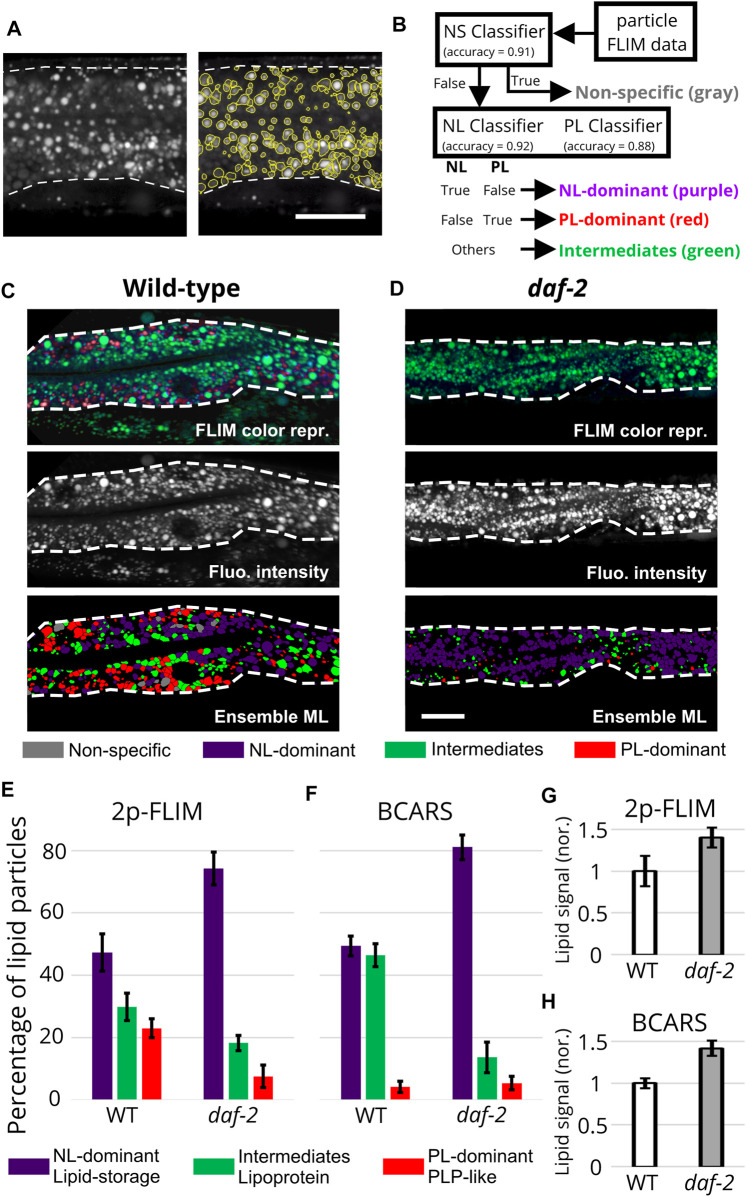
Classification of lipid particles imaged by Nile Red 2p-FLIM and label-free BCARS. **(A)** The results of marker-controlled watershed segmentation. **(B)** The classification pipeline of ensemble classification. NL, non-polar lipid; PL, polar lipid. **(C)**, **(D)** From upper to bottom, FLIM color representation, fluorescence intensity, and the results of ensemble classification for wild-type and *daf-2* 1-day adult worms. Scale bar, 20 *μ*m. **(E)**, **(F)** The comparison between 2p-FLIM and BCARS classification. **(G)**, **(H)** The normalized intestinal lipid signal obtained by NR-2p-FLIM after eliminating non-specific binding signal and BCARS 2845 cm^−^1 signals. Both results are the integrated signals divided by the area of the intestine. For Nile Red 2p-FLIM, data were obtained from *n* = 8 and 9 animals for wild-type and *daf-2*, respectively. About 4,200 (wild-type) and 5,900 (*daf-2*) lipid particles were analyzed. For BCARS, data were obtained from *n* = 5 animals for both wild-type and *daf-2*, and about 1,170 (wild-type) and 3,200 (*daf-2*) lipid particles were analyzed. Error bars represent the standard error of the mean.

To estimate the relative abundances of the different lipid particles in Nile Red 2p-FLIM images, we modified the previous ensemble machine-learning approach for BCARS imaging (Chen et al., 2020). We first applied a marker-controlled watershed algorithm (Parvati et al., 2009) to the fluorescence intensity image to define the boundary of each lipid particle. Figure 4A shows the results of intestinal lipid particle segmentation with the local maxima as the markers for watershed seeding. Once these objects were spatially defined, we calculated a 64-dimensional FLIM and morphological descriptor matrix for each particle (see *Methods* for details). Then, we used descriptors obtained from non-specific stained particles under low-concentration Nile Red staining, DHS-3::GFP-positive particles in early L4 worms, and VIT-2::GFP-positive PLPs to train three ensemble classifiers for classifying non-specific stained (NS classifier), non-polar lipid-dominant (NL classifier), and polar lipid-dominant (PL classifier) particles. Each classifier was an ensemble binary classifier (one-versus-rest) incorporating linear support vector machines, decision trees, logistic regression, K-nearest neighbors, and multi-layer perceptron neural nets, and the final classification results were determined by majority vote. About 6,000 particles in total were pooled for the classifier training process. The precision for the three ensemble classifiers was ∼90% (See section *Materials and Methods* for classification training details).

We next applied the three ensemble classifiers to each of 10^4^ lipid particles we imaged with 2p-FLIM in 1-day adult wild-type worms (Figure 4C) and *daf-2* (*e1370*) mutants (Figure 4D), a long-lived mutant that is frequently used in aging-related studies due to reduction of activity of the insulin-like receptor DAF-2 (Kimura et al., 1997; Kern et al., 2021). We found only about 3% of the particles were non-specifically stained. This indicates the marker-controlled watershed algorithm can effectively detect those lipid particles that were properly tagged by Nile Red based on their high-intensity contrast. The rest of the particles were then classified by both NL and PL classifiers. About 75% of Nile-Red-stained lipid particles were uniquely classified into either non-polar lipid-dominant or polar lipid-dominant categories. The remaining 25% lipid particles may contain various non-polar/polar lipid ratios in between lipid-storage and PLP components, essentially identical to the lipoprotein intermediates characterized by BCARS (Chen et al., 2020). Figure 4B summarizes the classification logic flow described above. We also examined wild-type and *daf-2* worms at the same 1-day adult stage with BCARS. The ensemble classification workflow described in reference (Chen et al., 2020) was applied to the collected BCARS data.

We next examined the relative abundance of the two particle types and intermediate mixtures in the intestine of wild-type and *daf-2* (*e1370*) 1-day adult worms imaged by Nile Red 2p-FLIM and label-free BCARS (Figures 4E, F; Supplementary Figure S7). In wild-type intestines, both 2p-FLIM and BCARS results show that nearly 50% of the particles are non-polar lipid-dominant, lipid-storage particles. The percentage of yolk-related particles (PLPs + lipoprotein intermediates) measured by both approaches are highly consistent ( ∼53% for 2p-FLIM and ∼51% for BCARS). The Nile Red 2p-FLIM results show fewer intermediates and more polar lipid-dominant particles compared to BCARS measurement. The difference is probably due to the lower chemical specificity of Nile Red, which can only differentiate environmental polarity. In contrast, BCARS collects all of the particle-related chemical information (Chen et al., 2020), such as protein band (phenylalanine ring breathing) at ∼1,002 cm^−1^, unsaturated carbon–carbon bonds at ∼1,650 cm^−1^, amide I resonance at ∼1,665 cm^−1^, ester carbonyl resonance (Raman marker band of triacylglycerols) at ∼1740 cm^−1^, lipid CH_2_ symmetric stretching at ∼2,845 cm^−1^, protein/lipid CH_2_ symmetric stretching at ∼2,925 cm^−1^, and thus yields more precise characterization. In the *daf-2* mutants’ intestines, similar results of particle percentage distribution were obtained by 2p-FLIM (74%, 18%, 8% for NL-dominant, intermediates, PL-dominant particle types, respectively) and BCARS (81%, 14%, 5% for lipid-storage, lipoprotein, PLP-like particle types, respectively). Finally, we compared the normalized lipid signal in the intestine of two strains (Figures 4G, H). We calculated integrated Nile Red fluorescence (excluding the non-specific stained particles) or lipid 2,845 cm^−1^ BCARS intensity of all intestinal lipid particles shown in each 2p-FLIM or BCARS image, and then normalized the integrated intensity by dividing the tissue area. Both 2p-FLIM and BCARS quantification results show about 1.4-fold increased intestinal lipid level in *daf-2* compared to the wild-type. These results are consistent with previously reported data, where the *daf-2* mutants show notable accumulation of fat detected by stimulated Raman scattering (Wang et al., 2011), reduced yolk production characterized by gel electrophoresis (Kern et al., 2021), and TAG/PL ∼ 2 in its lipid profile measured by chromatographic method (O’Rourke et al., 2009). Taken together, our results demonstrate that Nile Red 2p-FLIM is comparable with BCARS with respect to assessing *C. elegans*’ lipid particle sub-types and their abundance quantitatively.

## 4 Discussion

In this study, we demonstrated a novel Nile Red 2p-FLIM approach for discriminating lipid particle sub-types in live *C. elegans*. We showed that, with increased concentration, Nile Red vital staining is useful in detecting lipid-rich particles of *C. elegans*, solving the non-specific staining issue reported previously (O’Rourke et al., 2009; Yen et al., 2010). We found 4 h –8 h of 5 *μ*M Nile Red vital staining was sufficient to label a majority of lipid particles in the intestine, consistent with lipid intake timing measured by BCARS, where the appearance time of C-D signal in the worms fed with deuterated fatty acids is ∼4 h (Chen et al., 2020). Two-photon excitation for fluorescence imaging has the advantages of reduced photobleaching, minimal phototoxicity, and the ability to resolve detail at greater depths in living tissues, and thus is ideal for *C. elegans in vivo* imaging. Combined with FLIM phasor and ensemble machine-learning analysis, our Nile Red 2p-FLIM approach overcomes the limitations of techniques that rely on sample fixation and retains the advantages of microscopic techniques in preserving tissue and sub-cellular anatomy while providing a rigorous characterization of lipid particle sub-types. The improved specificity of Nile Red imaging using 2p-FLIM allows capturing the information on tissue-specific distribution, localization, and dynamics of different types of lipid-rich particles, and thus will be useful in addressing outstanding problems related to fat regulation and aging in the field (Ezcurra et al., 2018; Kern et al., 2021; Zhai et al., 2022).

While we have previously shown that BCARS can provide deeper characterization, Nile Red 2p-FLIM is more accessible since BCARS is not yet commercially available. Further, since the dimensionality of FLIM data is significantly lower than that of BCARS, the signal processing and analysis time of the former is much shorter. The Nile Red 2p-FLIM hardware platform reported here can be further improved to reach higher imaging speed by choosing a suitable laser pulse repetition rate and photon counting rate or using a wide-field microscope configuration (Oleksiievets et al., 2020; Sorrells et al., 2021), or to achieve a better spatial resolution that is close to super-resolution microscopy by replacing the single-element detector with a 2D SPAD detector array (Castello et al., 2019).

While Nile Red staining has been considered unreliable for identifying lipid structures in live *C. elegans*, our work demonstrates that highly reliable results of lipid structure imaging can be obtained from vital Nile Red staining at the concentration we employed when coupled with 2p-FLIM excitation at 920 nm and phasor analysis. In our work, we did not observe a strong Nile Red fluorescence signal in the non-specific staining regions with low-concentration Nile Red vital staining under the excitation of a 920 nm, 100 fs pulse. The mean intensity of those non-specific staining regions was about 5–10 times weaker than that in lipid-rich particles (Supplementary Figure S3). Yen et al. (2010) have reported that in the live worms fed with low-concentration Nile Red, the Nile Red signal was poorly colocalized with CARS lipid signal at 2,845 cm^−1^. These Nile Red molecules were likely trapped in the acidic gut granules and emitted a red-shifted fluorescence signal centered around 650 nm (Yen et al., 2010), an emission wavelength closer to the emission peak of Nile Red in water (657 nm) than that in polar lipids (620 nm) (Greenspan and Fowler, 1985). The red-shifting of Nile Red emission fluorescence wavelength has been characterized accompanied by a red shift of the whole excitation spectrum (about 45 nm red shifting under single-photon excitation) (Greenspan and Fowler, 1985). The weak fluorescence signal from the Nile Red molecules in the non-specific staining region we observed was likely due to a red shift in two-photon excitation wavelength, where the 920 nm, 100 fs pulses cannot strongly excite this population, essentially reducing the non-specific staining signals. Further, we have demonstrated that the non-specific staining cluster in phasor plots was clearly separated from those lipid staining signals (Figure 2D) and can be removed after the ensemble classification. Since the phasor analysis is independent of fluorescence signal intensity, similar analysis results can be obtained whether the non-lipid staining signal of Nile Red is effectively excited or not.

In summary, our results show that the Nile Red 2p-FLIM approach is comparable to label-free BCARS measurement with respect to quantitative estimation of lipid content and detection of lipid particle sub-types *in vivo*. The Nile Red 2p-FLIM offers a novel approach to examine living, intact specimens with imaging speed about 10× faster than BCARS, providing direct insight in the dynamics of lipid homeostasis. Our measurement protocol and analysis pipeline can be directly applied to other FLIM instruments such as frequency-domain FLIM (Raspe et al., 2016; Dunkers et al., 2021) or real-time pixel phasor displayed FLIM (Sorrells et al., 2021). Applying advanced deep learning algorithms such as U-Net convolutional neural network (CNN) (Smolen and Wooley, 2022) could possibly further improve the specificity and accuracy of Nile Red 2p-FLIM. Since the two-photon laser can simultaneously excite multiple dyes or fluorescent proteins, it is possible to study lipid/yolk interactions with other labeled proteins or organelles in live *C. elegans*. We expect this novel approach reported here to be used to examine tissue-resolved lipid/yolk signaling networks during aging.

## Data Availability

The original contributions presented in the study are included in the article/Supplementary Material, further inquiries can be directed to the corresponding authors.
