# Supplementary material for: Identifying lipid particle sub-types in live Caenorhabditis elegans with two-photon fluorescence lifetime imaging
Source: Front Chem. 2023 Apr 13;11:1161775. doi: 10.3389/fchem.2023.1161775 (PMC10137682; doi:10.3389/fchem.2023.1161775)
Supplement: Supplementary file 1 [file DataSheet2.pdf]

Supplementary Material

1 SUPPLEMENTARY FIGURES

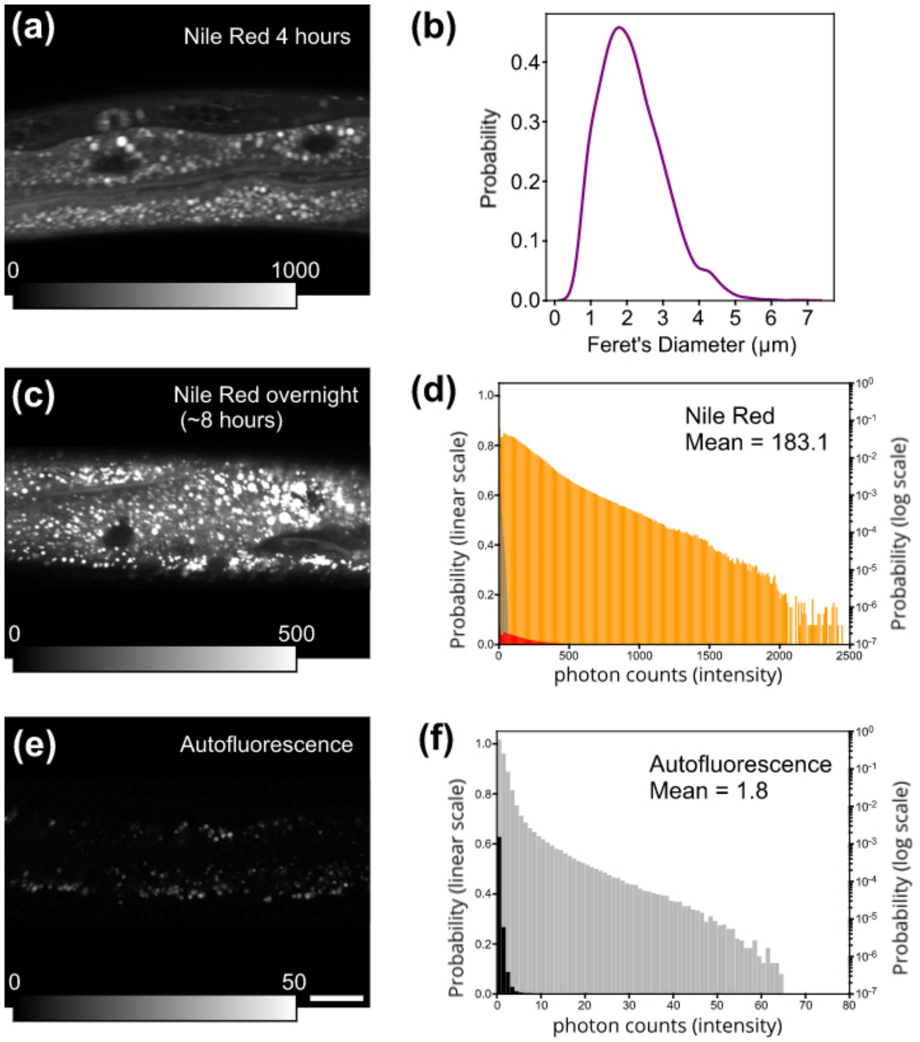

Figure S1. Comparison between Nile Red and autofluorescence signals. (a) A typical image of an adult wild-type worm with 4-hour, 5 μM Nile Red vital staining. The saturation gray level was set to 1000 counts. (b) The size distribution of lipid particles observed by Nile Red 2p-FLIM. Data obtained from n=4 biologically independent animals, about 2000 lipid particles. (c) A typical image of an adult wild-type worm with overnight 5 μM Nile Red vital staining. The saturation gray level was set to 500 counts. (d) The fluorescence intensity histogram of (c). Red for linear scale, orange for log scale. (e) A typical autofluorescence image of an adult wild-type worm. The saturation gray level was set to 50 counts. (f) The fluorescence intensity histogram of (e). black for linear scale, gray for log scale. The same gray histogram is also shown in (d). The pixels with intensity below 80 are not included for further phasor analysis. Scale bar, 20 μm.

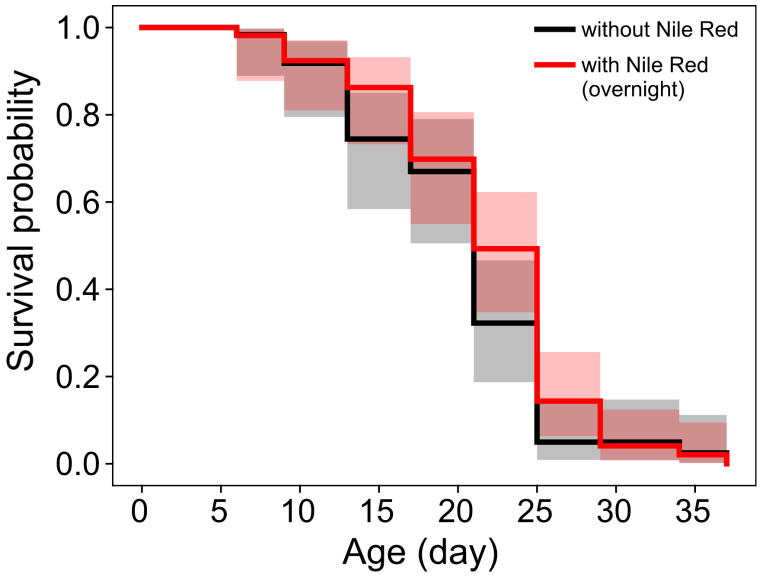

Figure S2. Comparison between the lifespan of wild-type worms with or without 5μM Nile Red overnight feeding. Log-rank test was performed for the comparison. The resulting chi-square test statistic value = 0.0284 (or p-value = 0.8662). The gray or light red area indicates the 95% confidence interval (n=90 for both conditions).

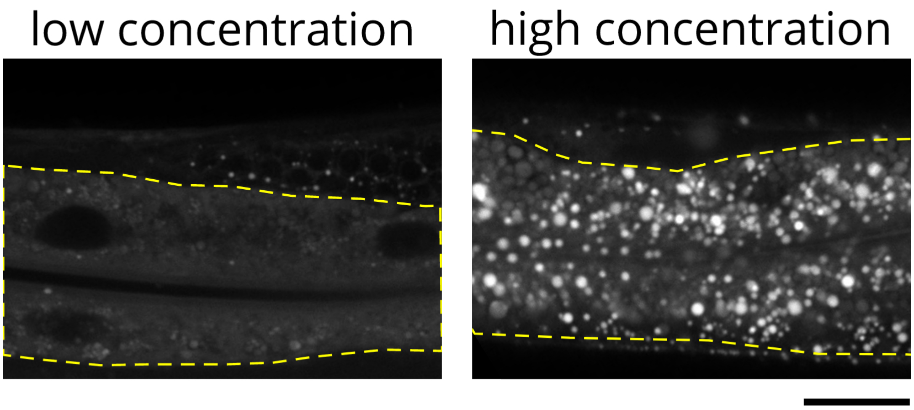

Figure S3. FLIM intensity images of wild-type adult worms with (left) low-concentration and (right) high-concentration Nile Red vital staining. Yellow dashed lines indicate intestine. Scale bar, 20 μm.

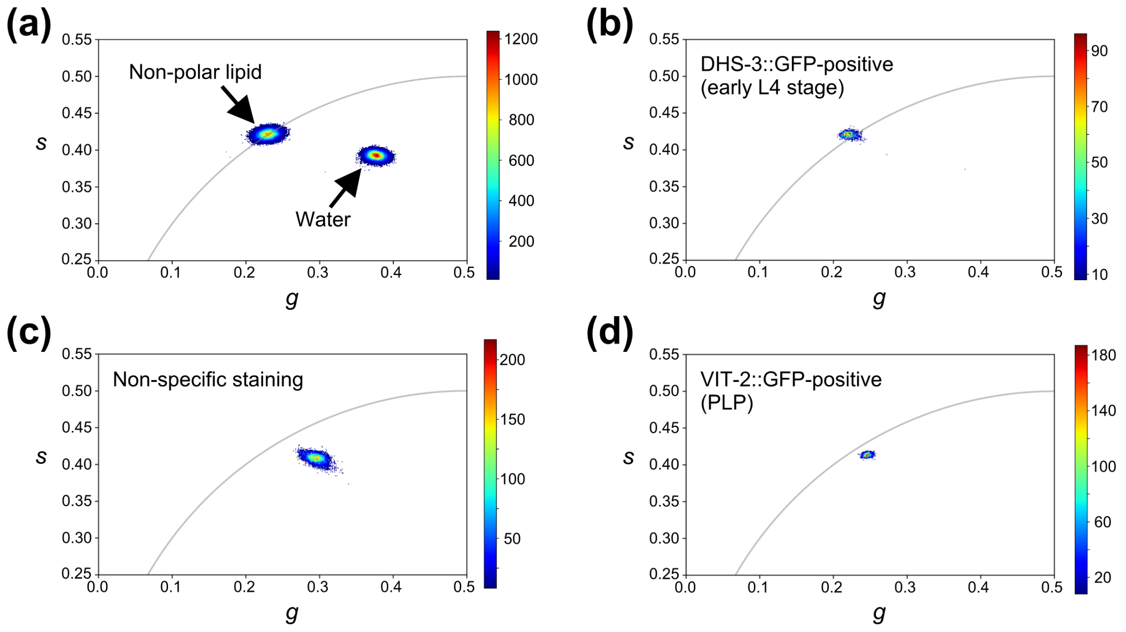

Figure S4. The phasor plots of Nile Red signal in (a) non-polar lipid solution and water, (b) DHS-3::GFP positive region in early L4 worms, (c) non-specific staining (low-concentration Nile Red vital staining), (d) VIT-2::GFP-positive region in PLP. The color bar represents the pixel counts.

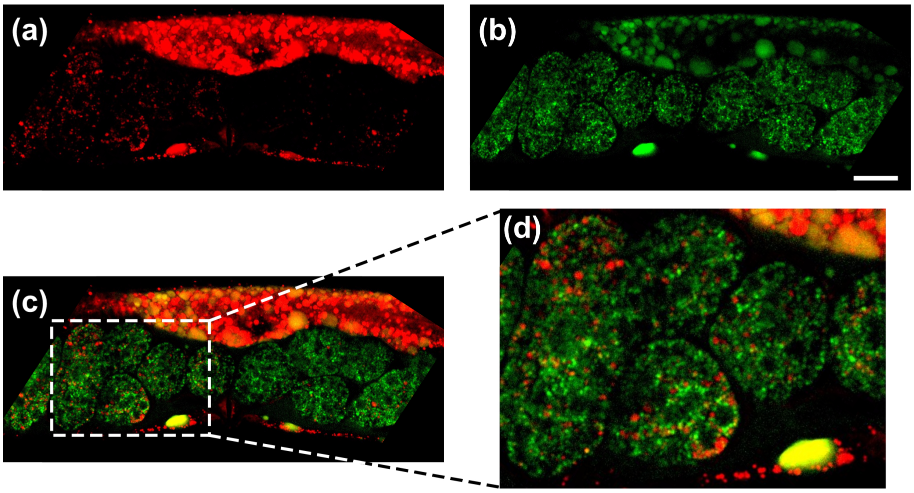

Figure S5. Delipidation of VIT-2::GFP-containing particles in embryos. (a) Nile Red, (b) VIT-2::GFP, and (c) merge images. (d) Expansion of the white box shown in (c). Scale bar, 20 μm.

htbp]

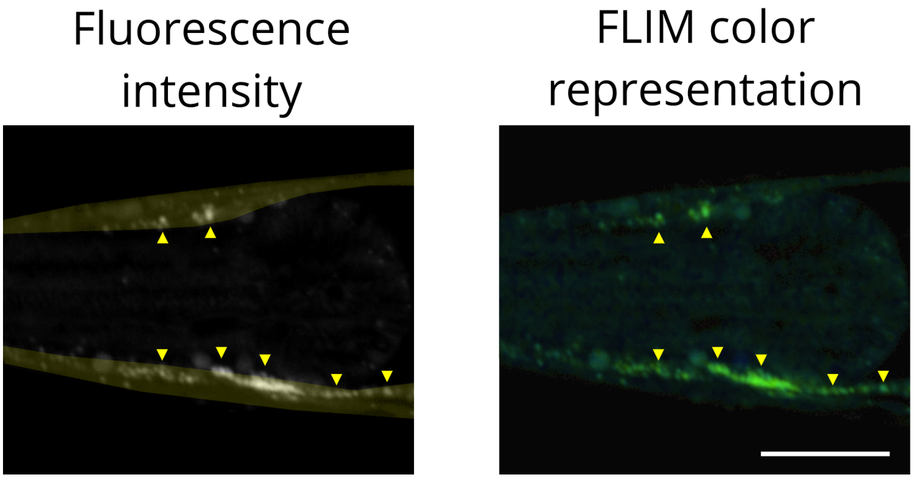

Figure S6. (left) Fluorescence intensity and (right) FLIM color representation of the skin-like epidermis near pharynx (yellow region) in a Nile-Red-stained wild-type adult worm. Some lipid-storage-like particles (green/yellow-green in the FLIM color representation image) are indicated with yellow arrowheads. Scale bar, 20 μm.

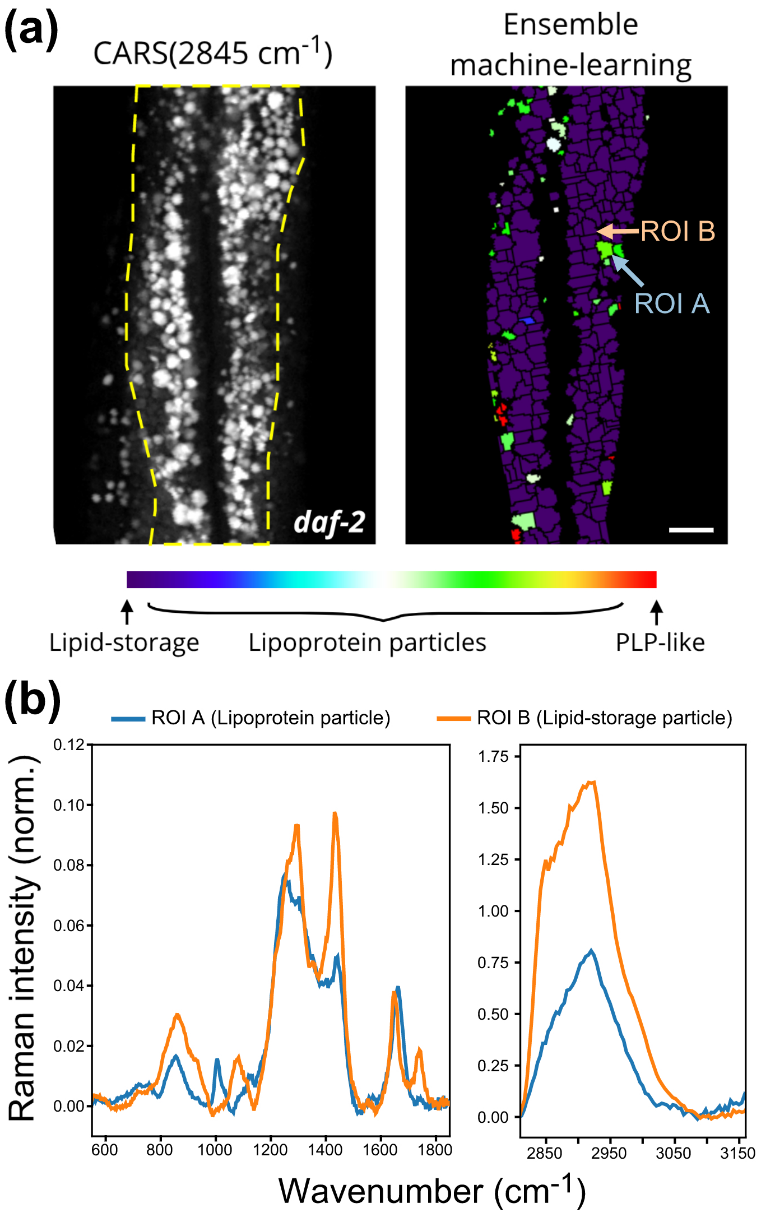

Figure S7. (a) BCARS 2845 cm^{-1} lipid image and the result of BCARS ensemble machine-learning analysis of unstained, *daf-2* adult worms. See reference (Chen et al., 2020) for the details of BCARS ensemble machine-learning analysis. Scale bar, 10μm. (b) The corresponding mean Raman spectra of the ROI A and ROI B indicated in (a).

REFERENCES

Chen, W.-W., Lemieux, G. A., Camp, C. H., Chang, T.-C., Ashrafi, K., and Cicerone, M. T. (2020). Spectroscopic coherent raman imaging of caenorhabditis elegans reveals lipid particle diversity. *Nature Chemical Biology* 16, 1087–1095. doi:10.1038/s41589-020-0565-2
