# Supplementary figures and images for: Identifying lipid particle sub-types in live Caenorhabditis elegans with two-photon fluorescence lifetime imaging
Source: Front Chem. 2023 Apr 13;11:1161775. doi: 10.3389/fchem.2023.1161775 (PMC10137682; doi:10.3389/fchem.2023.1161775)

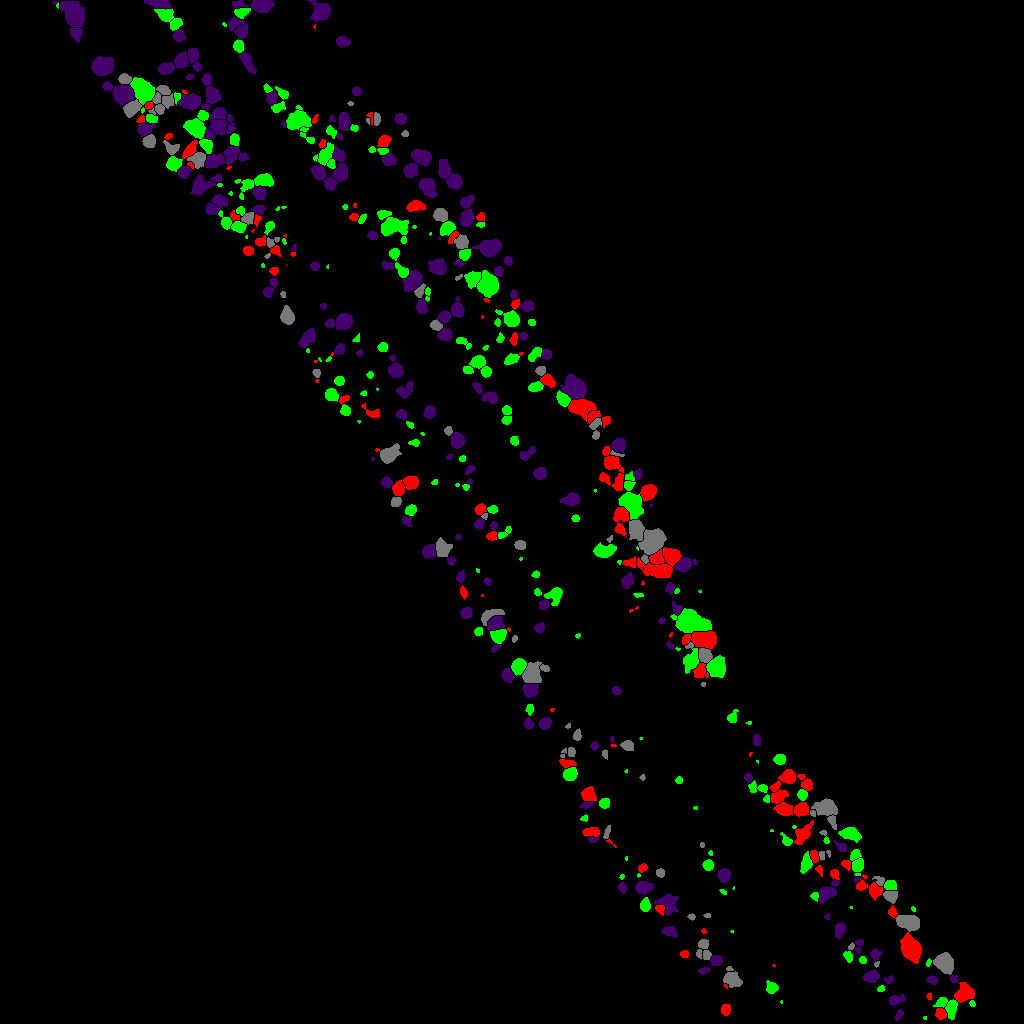

Supplement: Supplementary file 2 [file DataSheet1.ZIP › particle_segmentation/sample_image(Classification_results).png]

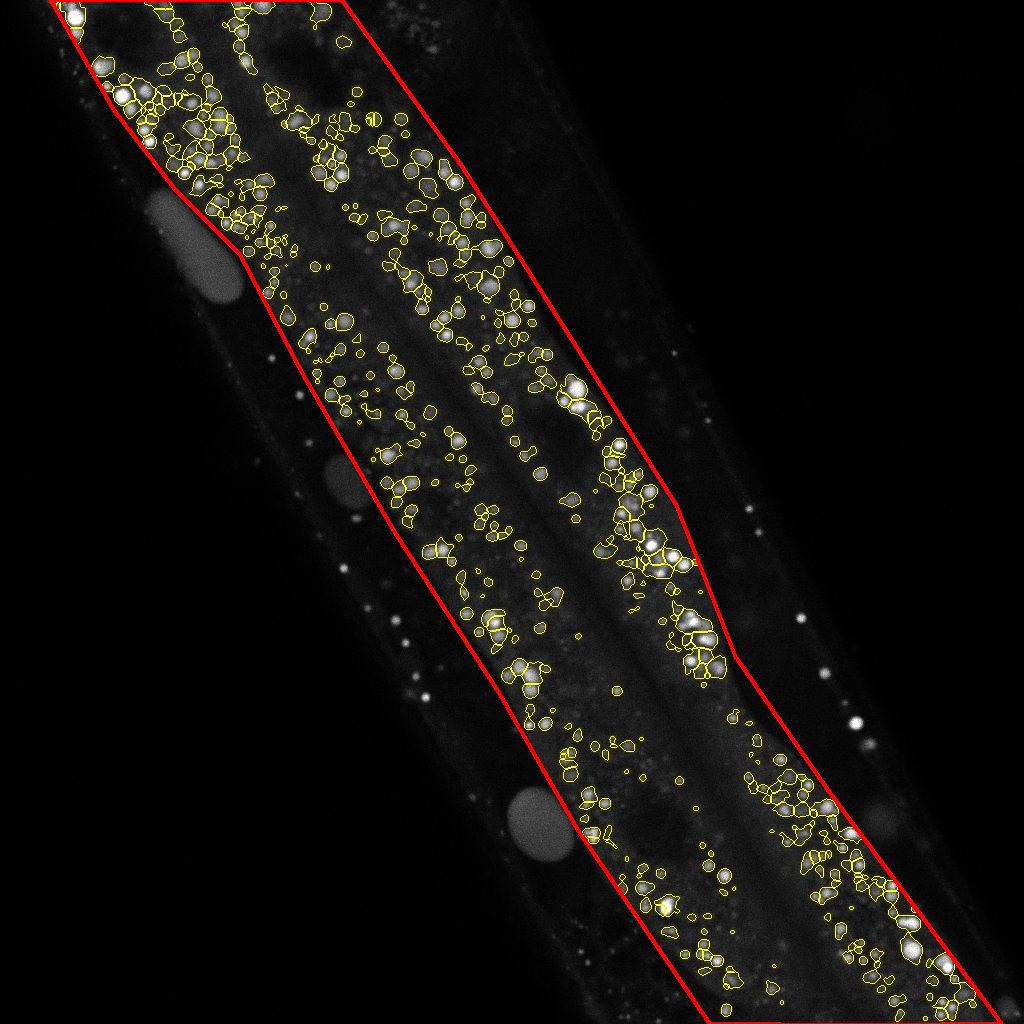

Supplement: Supplementary file 2 [file DataSheet1.ZIP › particle_segmentation/sample_image(particles_intestine_boundary).jpg]
